# Impact of patient motion on parametric PET imaging

**DOI:** 10.1007/s00259-024-06599-9

**Published:** 2024-01-15

**Authors:** Alessia Artesani, Joyce van Sluis, Johannes H. van Snick, Laura Providência, Walter Noordzij, Charalampos Tsoumpas

**Affiliations:** 1 Department of Biomedical Sciences, Humanitas University, Via Rita Levi Montalcini 4, 20072 Pieve Emanuele, Italy; 2 Department of Nuclear Medicine and Molecular Imaging, University Medical Center Groningen, University of Groningen, Hanzeplein 1, 9713 GZ Groningen, Netherlands


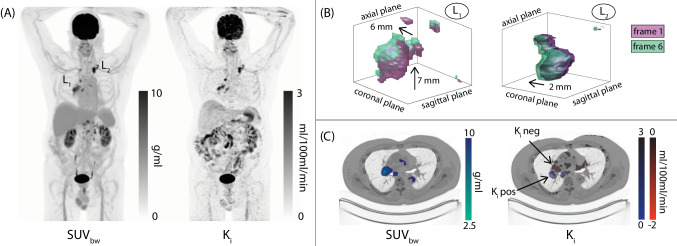


A 64-year-old male patient, newly diagnosed with Hodgkin lymphoma, underwent dynamic total-body [^18^F]FDG PET/CT imaging. The PET list mode data was binned into frames, and the tomographic images were reconstructed following a previously published protocol [1]. The net influx rate (*K*_*i*_) image was obtained from dynamic PET images by applying the Patlak graphical method, and subsequently, it was compared with the standardized uptake value image normalised to body weight (A). The patient displayed hypermetabolic lymph nodes in the mediastinum (referred to as lesion L_1_) and in the left lung (L_2_). Upon comparison between PET frames, a misalignment in the lung position, and consequently in the lesion positions, were observed and ultimately associated with the patients’ respiratory motion patterns. The largest misalignment was observed between the first and last frames, and the corresponding frame overlap is depicted in Figure (B). The position of the lesion *L*_1_ across the PET frames varied, amounting to approximately 6–7 mm along the coronal and axial plane (B-left), while a modest movement of approximately 2–3 mm was detected for lesion L_2_ (B-right). These mismatches in the lesion position throughout the acquisition window led to inaccurate parametric net influx rate assessments. The *K*_*i*_ image showed (i) a reduced volume of the L_1_ lesions (Δ*V* = 3.3 cm^3^, − 15%) and (ii) the emergence of negative *K*_*i*_ values, primarily within the regions most affected by motion (C). The *K*_*i*_ image artefacts compromise the accuracy of tumour metabolic rate evaluation, carrying significant clinical implications, especially within the domain of oncology [2]. Considering the expanding role of parametric analysis in clinical practice [3, 4], the effective identification and correction of artefact sources in parametric image data represent a central challenge in the translation of this research into clinical application.

## Data Availability

The datasets generated during and/or analysed during the current study cannot be available due to ethics restrictions.
